# Prioritising communicable disease research in Afghanistan: an application of the Child Health and Nutrition Research Initiative (CHNRI) methodology

**DOI:** 10.1136/bmjgh-2025-020891

**Published:** 2026-05-19

**Authors:** Emily C Keats, Tanvi Majumdar, Curie Kim, Sama El Baz, Hana Tasic, Robert E Black, David H Peters, Najibullah Safi, Hannah Tappis, Kerri Wazny, Nadia Akseer, Afghanistan Communicable Disease Research Prioritization Collaborators, A Ahmadi

**Affiliations:** 1 Johns Hopkins Bloomberg School of Public Health, Baltimore, Maryland, USA; 2 Modern Scientist Global, St. Catharines, Ontario, Canada; 3 School of Global Health, York University, Toronto, Ontario, Canada; 4 Independent Consultant, Kabul, Afghanistan; 5 Jhpiego, Baltimore, Maryland, USA

**Keywords:** Communicable Diseases, Emerging, Global Health, Child health, Vaccines

## Abstract

**Introduction:**

Communicable disease control in Afghanistan has deteriorated amid growing fragility, health system disruption and declining international aid since the 2021 regime change. Outbreaks of measles, pertussis, pneumonia, cholera, malaria, dengue, Crimean-Congo haemorrhagic fever, tuberculosis and polio continue to plague the population in Afghanistan. This study addresses a critical evidence gap by systematically ranking research priorities for communicable diseases in Afghanistan.

**Methods:**

This study applied the Child Health and Nutrition Research Initiative (CHNRI) methodology, which is a widely used approach for systematic, transparent and collaborative research priority setting. It leverages expert consultation to generate, score and rank research questions. This study identified and invited 303 Afghanistan-health researchers, based globally, to complete the survey which consisted of 33 research questions related to communicable diseases that were submitted by 15 researchers.

**Results:**

This CHNRI exercise included 44 respondents, 63.6% of whom were of Afghan origin. The top 10 highest-ranked questions focused on identifying barriers to low measles and polio vaccination coverage, assessing disease burden by region and strategies to reduce the incidence of tuberculosis. Respondents of Afghan origin ranked antibiotic resistance and gender-related disparities in tuberculosis as the highest-priority questions. The majority of priority questions were description questions.

**Conclusions:**

Researchers, governments, donors, policy makers and programme implementers can use these findings as a starting point to strategically align research agendas, guide resource allocation, and prioritise evidence-based interventions for life-saving communicable disease prevention and control in Afghanistan.

**WHAT IS ALREADY KNOWN ON THIS TOPIC**

**WHAT THIS STUDY ADDS**

- To the authors’ knowledge, this global study is the first to apply the Child Health and Nutrition Research Initiative methodology to communicable disease research in Afghanistan, identifying and ranking the top 10 priority questions.

**HOW THIS STUDY MIGHT AFFECT RESEARCH, PRACTICE OR POLICY**

- The findings of this study will serve as a starting point for broader discussions related to investing in research to address the burden of communicable diseases in Afghanistan. Answering priority research questions will inform policy and programming decisions, ultimately having positive impacts on the health and well-being of the Afghan population.

## Introduction

 Communicable disease management in Afghanistan faces serious challenges due to longstanding political and economic instability and recurrent environmental disasters.^1^,^3^ Since 2021, the combined effects of heavy donor dependency with declining international aid, critical shortages of health workers, loss of vaccination infrastructure and weakened communicable disease surveillance systems have severely undermined efforts to prevent and control communicable diseases.^2 3^ Afghanistan’s humanitarian crisis, compounded by worsening natural disasters due to climate change, have further increased the risk of waterborne diseases, food insecurity, malnutrition and population displacement, which facilitate the transmission of communicable diseases.^4^ In addition, immunisation levels for vaccine-preventable diseases are low across Afghanistan, leading to outbreaks.^5^

Afghan communities face a high incidence of preventable diseases including measles, pneumonia, diarrhoeal disease, malaria, wild poliovirus, tuberculosis (TB), dengue and Crimean-Congo haemorrhagic fever.^6^,^8^ WHO surveillance showed over 55 000 suspected measles cases and 357 related deaths from December 2024 to May 2025.^7^ During that period, only 51% of eligible children had received their first dose of the measles vaccine, and just 37% had received their second dose.^9^ While childhood vaccination levels have always been low in Afghanistan, they have worsened in recent years due to the erosion of infrastructure.^5^

With poorly functioning health facilities, preventable disease outbreaks are widespread. More than 2000 health facilities faced a risk of closure in 2021.^10^ By mid-2025, 80% of WHO-supported essential healthcare services were at risk of shutdown, and approximately 400 health facility closures had cut-off access to preventive care for over 3 million Afghans.^9^

Afghanistan’s path to preventing avoidable deaths and achieving the United Nations 2030 Sustainable Development Goals (SDGs) requires strategic, evidence-based resource allocation and sustained advocacy. With limited funding and operational capacity, it is essential to identify the highest priority communicable disease research questions to guide high-impact research agendas and efficient use of resources. Aligning efforts across key stakeholders can strengthen interventions in this fragile context.

This paper applies the Child Health and Nutrition Research Initiative (CHNRI) methodology to set short- to medium-term priorities for communicable disease research in Afghanistan to align with the 2030 SDG agenda. A 2024 research priority discussion series, conducted by the Afghanistan MoPH with support from the WHO, included the communicable disease topic area, among others. However, this CHNRI does not incorporate or report on those discussions, which are not publicly available. Other CHNRIs have been conducted on (1) health systems and (2) maternal and child health, sexual and reproductive health and nutrition in Afghanistan and their findings have been published as separate papers within this Supplement.^11 12^ The findings from this CHNRI will enable researchers, governments, donors, academic institutions, policy makers and programme implementers to make informed decisions to address the communicable disease crisis in Afghanistan.

## Methods

To identify health research priorities in Afghanistan, we applied the CHNRI methodology—a widely used approach that enables transparent and systematic prioritisation through expert consultation and by scoring research questions against predefined criteria.^13^,^17^ The process unfolded in four distinct stages, outlined below and described in detail in prior publications.^11 12^ The complete research process took place over several years, spanning from 2022, when experts were identified and research questions were generated, to 2025, when the survey was distributed and results were analysed.

### Strategic planning and criteria definitions

An advisory group of Afghan and international experts, on health research in Afghanistan and/or the CHNRI process, was assembled to define the scope and criteria of the exercise (online supplemental table 1) along with the core research team at Johns Hopkins University. Based on previous CHNRI applications,^18^ 13 candidate criteria for scoring research questions were presented to the advisory board for ranking; these incorporated the five ‘standard’ CHNRI criteria (answerability, effectiveness, deliverability, maximum potential for disease burden reduction and the effect on equity) and eight others that are applicable for research in the Afghanistan context (online supplemental table 2). The five highest-ranked criteria were then selected and tailored to the Afghan context through corresponding guiding questions.

The five selected CHNRI criteria included: (1) Feasibility—is it feasible to conduct this research considering cultural, financial, human resources, logistical and security challenges? (2) Effectiveness—would interventions arising from the proposed research improve health outcomes in Afghanistan? (3) Equity—would answering the question enable interventions that reduce population inequities? (4) Answerability—is it possible to design an ethically sound and implementable research study that can credibly answer the question? (5) Disease burden reduction—would interventions stemming from this research contribute to a significant reduction in mortality and/or morbidity across the country?

### Researcher engagement and question development

To identify experts in health and health research in Afghanistan, researchers with a publication record on health in Afghanistan (3+relevant articles from 2005 to 2022) were identified via PubMed and Scopus. Eligible individuals (N=303) were contacted and invited to propose up to three priority research questions, which were submitted to the research team via email. In total, 96 researchers submitted 323 research questions that were then grouped by topic area and refined through a multi-stage synthesis process, including the use of AI support (ChatGPT V.3.5) to consolidate related questions. For example, submitted questions that were similar were provided to ChatGPT with instructions to combine them. The study team then reviewed and edited the output to produce a final, synthesised question that was clear and captured the intent of the original submissions. This process resulted in a final list of 33 questions within the communicable disease topic area. These 33 questions were submitted by only 15 researchers. Questions outside the communicable disease scope were routed to two parallel CHNRI exercises examining health systems and maternal and child health in Afghanistan^11 12^ ; it was at this point in the process where the three CHNRI exercises diverged, having shared the strategic planning, criteria definition, researcher engagement, and question development phases.

The final 33 questions were categorised into the CHNRI 4D framework.^18^ In this framework, proposed research falls into one of the four domains: (1) description—research to assess the burden of the health issue and its determinants, (2) delivery—research to optimise health through existing means (ie, implementation research), (3) development—research to improve existing health interventions or (4) discovery—research that leads to new innovations.

### Survey implementation and scoring

To score the final communicable disease research questions against the criteria, a survey was developed using REDCap and distributed to all eligible researchers (n=303), regardless of whether they had submitted a research question. Participants rated each question across the five predefined criteria using a standardised response format. Participation reminders and question randomisation were used to enhance data completeness and reduce bias. Researchers were given 6 weeks to complete the ranking exercise (including a 2-week extension), and group reminders were sent weekly. After 4 weeks, individual reminders were also sent. Participation was fully voluntary and respondents were not reimbursed.

It should be noted that the 303 eligible researchers were invited to participate in all three CHNRI topics (communicable diseases, health systems and maternal and child health), but that the surveys were sent at different time points that spanned a year between the health systems and maternal and child health topics and 4 months between the maternal and child health and communicable disease topics. Participation in each case was through self-selection.

### Analysis and prioritisation

Data were analysed using equal weighting across criteria, based on agreement among the advisory board. Two primary metrics were computed: the Research Priority Score (RPS), indicating the average level of expert endorsement, and the average expert agreement (AEA) score, reflecting consensus. The RPS is a mean score, ranging from 0% to 100%, calculated for each individual research question per criterion as well as overall (across all five criteria) for each question. This is an average of participants’ responses of ‘yes’ (1 point), ‘no’ (0 points) or ‘undecided’ (0.5 points). The higher the RPS, the higher the question’s priority rank. The AEA assesses the proportion of scorers who selected the most common score (mode) for each research question. An AEA of 75% and above indicates a high level of agreement.

Results were analysed overall and by those who identified as Afghan origin versus non-Afghan origin respondents to descriptively compare potential differences in research priorities.

All analysis was conducted with Stata V.18.5.

The REporting guideline for PRIority SEtting of health research (REPRISE) was used to support reporting and is found as online supplemental material.

## Results

### Characteristics of the respondents

Table 1 details the demographic characteristics of survey respondents. Of the 44 participants, 33 completed all the questions. The response rate was 14.5%, as the survey was sent to all identified researchers (n=303). Given the time lapse between question generation and survey distribution, it is likely that some of these individuals were not reached via email. Only 15 researchers submitted communicable disease-relevant research questions during the question development phase, indicating that the proportion with expertise in this area is likely to be limited when compared with the other CHNRI topics (60 researchers submitted health systems questions and 53 submitted questions on maternal, newborn and child health, nutrition and sexual and reproductive health).^11 12^ Of the 15 who submitted questions, 7 also responded to the survey. Analyses were conducted on the entire sample (N=44) to preserve representativeness, maximise use of available data and assess potential biases associated with nonresponse.

**Table 1:** Demographic characteristics of survey respondents (n=44)

Characteristic	Proportion n (%)
Afghan origin	28 (63.6)
Non-Afghan origin	16 (36.4)
High-income country	25 (56.8)
Low or middle-income country	19 (43.2)
Men	36 (81.8)
Women	8 (18.2)
Years of experience in health research	
1–3 years	1 (2.3)
4–7 years	5 (11.4)
8–10 years	9 (20.5)
11–15 years	7 (15.9)
16–20 years	6 (13.6)
20+	16 (36.4)
Years of health research experience related to Afghanistan	
1–3 years	6 (13.6)
4–7 years	6 (13.6)
8–10 years	13 (29.6)
11–15 years	7 (15.9)
16–20 years	6 (13.6)
20+	6 (13.6)
Area of expertise (self-identified, non-exclusive)*	
Health systems	30 (68.2)
Communicable diseases	29 (65.9)
Maternal, neonatal and child health	27 (61.4)
Noncommunicable diseases	18 (40.9)
Nutrition	17 (38.6)
Sexual and reproductive health	10 (22.7)
Mental health and substance use	6 (13.6)
Disability and injuries	0 (0)
Other	8 (18.2)
Place of employment	
Research/academia	25 (56.8)
Non-governmental organisation	16 (36.4)
Healthcare professional	8 (18.2)
Governmental agency	4 (9.1)
Donor agency	0 (0)
Other	10 (22.7)

* Respondents could have selected more than one option.

About 64% of all respondents were of Afghan origin, despite the fact that more than half (57%) now reside in high-income countries. The vast majority (82%) were men. Participants reported varied years of experience in health research and health research relating to Afghanistan, with health systems most frequently cited as an area of expertise. It is worth noting that 29 out of 44 participants (66%) self-identified as communicable disease experts. More than half of respondents (57%) work in research or academia, while just over a third (36%) work for non-governmental organisations (NGOs) and fewer work as a healthcare professional (18%), in a government agency (9%) or other. No donors responded to this survey.

### Top research questions (overall)

The top 10 research priority questions based on the overall RPS and AEA are presented in table 2. Scores for RPS ranged from 89% to 93%, with AEA ranging from 81% to 89%, indicating high levels of consensus among experts. Most of the top 10 questions (60%) were description questions*.* Development and delivery questions each comprised 20%. The full set of ranked questions can be found in online supplemental table 3.

**Table 2:** Overall rank, intermediate Research Priority Score (RPS), overall RPS and average expert agreement (AEA) scores for the top 10 research questions overall

Research question (domain)	Feasibility	Effectiveness	Equity	Answerability	Disease burden reduction	Overall RPS	AEA
1. What are the barriers contributing to low polio and measles vaccination coverage among children in Afghanistan? (Description*)*	0.90	0.92	0.93	0.96	0.93	92.86%	0.86
2. What are the current burdens of infectious diseases, including vaccine-preventable diseases (eg, measles, polio, acute viral hepatitis, typhoid fever, pneumonia, meningitis), respiratory infections (eg, tuberculosis, pneumonia) and re-emerging diseases (eg, acute watery diarrhoea, measles, malaria), at the national and sub-national levels, and in rural areas of Afghanistan? (Description*)*	0.94	0.91	0.93	0.93	0.92	92.68%	0.89
3. What strategies should be considered to reduce the tuberculosis incidence in Afghanistan? (Development*)*	0.95	0.89	0.91	0.92	0.88	91.05%	0.85
4. How does the misuse of antibiotics, including overprescribing and self-medicating, affect antibiotic resistance and the effectiveness of infectious disease control measures in Afghanistan? (Description*)*	0.86	0.92	0.86	0.97	0.91	90.42%	0.87
5. What is the prevalence and associated morbidity and mortality of vaccine-preventable diseases among children in Afghanistan who are seeking health services from outpatient and inpatient departments at health facilities? (Description*)*	0.93	0.89	0.87	0.89	0.92	90.29%	0.84
6. What is the status of the immunisation programme and how can programme performance be improved to increase coverage under the new regime? (Delivery*)*	0.89	0.90	0.90	0.89	0.92	90.07%	0.81
7. What are the determinants of the higher prevalence of tuberculosis among women in Afghanistan compared with men? (Description*)*	0.84	0.91	0.94	0.89	0.92	90.07%	0.81
8. How can immunisation programmes and other strategies for reducing the prevalence and mortality of communicable and infectious diseases be effectively delivered across Afghanistan? (Delivery*)*	0.92	0.90	0.90	0.88	0.87	89.32%	0.83
9. What are the prevalence and risk factors of blood-borne diseases (eg, hepatitis B, hepatitis C, HIV/AIDS) at the national level in Afghanistan and among high-risk populations such as people who inject drugs, people living under the poverty line, the unemployed and internally displaced people? (Description*)*	0.80	0.93	0.94	0.90	0.88	88.91%	0.82
10. What are the most effective methods for identifying areas in Afghanistan with low polio and measles vaccination coverage? (Development*)*	0.88	0.92	0.84	0.91	0.90	88.87%	0.83

The overall RPS is the average score across participants (n=44) and criteria, where ‘yes’=1 point, ‘no’=0 points and ‘undecided’=0.5 points. An AEA >0.75 indicates a high level of agreement between respondents.

The highest ranked research question focused on identifying barriers contributing to low polio and measles vaccination coverage among children in Afghanistan (RPS: 92.9%, AEA: 86.3%). Other highly ranked questions included assessing the current burden of infectious diseases nationally, subnationally and in rural settings (RPS: 92.7%, AEA: 88.8%) and identifying strategies to reduce TB incidence (RPS: 91.1%, AEA: 84.6%).

Most top-ranked questions were concentrated around immunisation and infectious disease control for vaccine-preventable diseases and TB. Several questions focusing on programme delivery and implementation challenges (eg, immunisation programme performance, effective delivery of immunisations and methods to identify areas of low vaccination coverage). Questions on antibiotic resistance and its role in infectious disease control, blood-borne disease prevalence and risk factors, and determinants of sex differences in TB prevalence among adults also featured prominently in the top ten.

### Comparison by subgroup: Afghan-origin versus non-Afghan-origin respondents

Table 3 shows the top 10 research priority questions for Afghan-origin experts. Among Afghans, the highest-ranked question was on the misuse of antibiotics and its effect on resistance and disease control (RPS: 96.1%). Questions on barriers to vaccination coverage, specifically measles and polio vaccines (RPS: 96.0%), determinants of higher TB prevalence among women (RPS: 93.2%) and effective delivery of immunisation programmes (RPS: 93.1%) also ranked prominently. Compared with the overall ranking (table 2), Afghan respondents placed greater emphasis on antimicrobial resistance and gender-related TB disparities, though most of the same research questions appeared in both top-10s. The full set of ranked questions among Afghan respondents can be found in online supplemental table 4.

**Table 3:** Overall rank, intermediate Research Priority Scores (RPS) and overall research priority scores for the top 10 research questions among Afghan-origin respondents

Research question (domain)	Feasibility	Effectiveness	Equity	Answerability	Disease burden reduction	Overall RPS
1. How does the misuse of antibiotics, including overprescribing and self-medicating, affect antibiotic resistance and the effectiveness of infectious disease control measures in Afghanistan? (Description)	0.93	1	0.93	1	0.95	96.12%
2. What are the barriers contributing to low polio and measles vaccination coverage among children in Afghanistan? (Description)	0.95	0.96	0.96	0.98	0.95	96.01%
3. What are the determinants of the higher prevalence of tuberculosis among women in Afghanistan compared with men? (Description)	0.90	0.93	0.93	0.93	0.98	93.24%
4. How can immunisation programmes and other strategies for reducing the prevalence and mortality of communicable and infectious diseases be effectively delivered across Afghanistan? (Delivery*)*	0.95	0.93	0.93	0.91	0.93	93.05%
5. What is the status of the immunisation programme and how can programme performance be improved to increase coverage under the new regime? (Delivery)	0.88	0.98	0.93	0.93	0.93	92.62%
6. What is the prevalence and associated morbidity and mortality of vaccine-preventable diseases among children in Afghanistan who are seeking health services from outpatient and inpatient departments at health facilities? (Description)	0.98	0.90	0.88	0.92	0.94	92.33%
7. What are the prevalence and risk factors of blood-borne diseases (eg, hepatitis B, hepatitis C, HIV/AIDS) at the national level in Afghanistan and among high-risk populations such as people who inject drugs, people living under the poverty line, the unemployed and internally displaced people? (Description)	0.85	0.96	0.94	0.94	0.90	91.85%
8. What are the current burdens of infectious diseases, including vaccine-preventable diseases (eg, measles, polio, acute viral hepatitis, typhoid fever, pneumonia, meningitis), respiratory infections (eg, tuberculosis, pneumonia) and re-emerging diseases (eg, acute watery diarrhoea, measles, malaria), at the national and sub-national levels, and in rural areas of Afghanistan? (Description)	0.89	0.93	0.91	0.94	0.92	91.73%
9. How can leishmaniasis cases best be identified and followed-up at the community level in Afghanistan? (Discovery)	0.93	0.90	0.92	0.95	0.89	91.68%
10. What strategies should be considered to reduce the tuberculosis incidence in Afghanistan? (Development)	0.98	0.88	0.86	0.90	0.88	89.86%

The overall RPS is the average score across participants (n=28) and criteria, where ‘yes’=1 point, ‘no’=0 points and ‘undecided’=0.5 points. An AEA >0.75 indicates a high level of agreement between respondents.

AEA, average expert agreement.

Non-Afghan-origin experts prioritised questions on TB and infectious disease burden, with the top-ranked question focusing on strategies to reduce TB incidence (RPS: 93.3%). Other highly ranked priorities included infectious disease surveillance, impact of displacement on global spread of infections and barriers to polio and measles vaccination. The full set of ranked questions among non-Afghan respondents can be found in online supplemental table 5.

While there was broad agreement between Afghan and non-Afghan respondents regarding the importance of vaccination coverage and TB control, the order of ranking differed. Afghan respondents emphasised antibiotic resistance and gender-related TB disparities, while non-Afghans gave higher priority to population displacement and its role in global infection spread. Both groups highlighted programmatic and delivery-focused research questions, reflecting consensus that actionable, implementation-oriented research is urgently needed.

## Discussion

To the best of our knowledge, this is the first CHNRI exercise centred on communicable diseases in Afghanistan. The 10 highest priority questions focused on barriers to immunisation, assessing disease burdens (eg, measles, polio, TB) and immunisation rates, and addressing implementation challenges to improve communicable disease management. Most of the top 10 questions were description questions, underscoring a need to first understand the situation of communicable diseases in Afghanistan before improving on or enacting new strategies to manage them. Findings from this CHNRI further demonstrate that the CHNRI methodology is useful for diverse applications in different settings.

The results of this CHNRI align closely with the realities of communicable disease management in Afghanistan, as concerns regarding immunisation rates and TB control remain among the most urgent health priorities. For instance, despite national polio vaccination campaigns, polio persists, indicating challenges in immunisation implementation and surveillance.^6^ In Afghanistan, door-to-door or mosque-to-mosque vaccination for children is supposed to be done by women, but growing restrictions on female workers and mobility have limited the reach of at-home vaccination campaigns.^6^ This, coupled with mistrust of vaccination campaigns and security concerns by the Taliban (in terms of revealing their location) which has led to the banning of door-to-door campaigns throughout Afghanistan, means that few children are being reached through this critical channel.^19^ Additionally, the 2023–2024 influx of a large number of Afghan returnees from Pakistan, where polio also persists, created uncertainty in population estimates for polio.^6^ A 2025 national measles immunisation campaign in Afghanistan, under the National Expanded Programme on Immunisation and supported by Gavi, reflects the country’s commitment to improve immunisation rates for preventable, fatal diseases.^20^ Before the campaign, only 55% of children aged 10 years and under had received their first measles dose.^20^ In addition to this, major inequalities in coverage by region have been noted, with 86% of children in Bamyan province having received all basic vaccinations compared with 5% and 3% in Nooristan and Urozgan, respectively.^21^

The highest priority questions among Afghan CHNRI respondents demonstrated an emphasis on gender-related TB disparities and antimicrobial resistance. Afghanistan has a high burden of TB particularly among women, impacted by increasingly limited access to healthcare for women, poverty, and fragile disease monitoring and reporting systems.^22^ Without strong surveillance and regulatory infrastructure in the healthcare system, antimicrobial resistance (AMR) is also difficult to detect and address.^23^ Unregulated antibiotic sales in Afghanistan, combined with limited awareness of AMR among patients, some healthcare providers and pharmacists, are associated with high AMR rates.^23^ However, the relative contribution of over-the-counter sales versus the other drivers (incomplete treatment courses, livestock antibiotic use, inadequate infection control in healthcare settings) to AMR burden remains poorly quantified in the Afghan context, representing a critical knowledge gap. It’s been estimated that AMR caused 5690 deaths in Afghanistan in 2021 alone (ie, those that could have been prevented without bacterial drug-resistance causing those deaths) while 21 900 deaths were associated with AMR over the same period (ie, those that would not have occurred had the infection been prevented), highlighting the urgency of this situation.^24^

Most of the top 10 questions in this CHNRI application were description questions, aligning with the critical need to improve national surveillance of communicable diseases and management programmes.^6 22 23^ With severe funding, public health capacity and logistical constraints to conducting research in Afghanistan, the likelihood of having discovery questions in the top 10 is very low. While these challenges could conceivably apply to any type of research in Afghanistan, there are existing local research organisations, academic institutions and NGOs that have the knowledge and capacity to undertake national and subnational surveys and qualitative research that could answer priority description questions pending requisite funding. The high proportion of description questions is similar to a sexual and reproductive health-focused CHNRI in Bangladesh,^25^ highlighting the need to undertake situational analyses before considering how to design or deliver interventions effectively in some low- and middle-income country (LMIC) contexts. CHNRIs done in other LMICs have also ranked immunisation-related questions highly, especially related to TB and strategies to improve vaccination coverage.^25^,^27^ In the related CHNRI on health systems in Afghanistan, the highest-ranking question was related to addressing health system barriers to immunisation uptake, further supporting the relevance of the findings from this CHNRI and underscoring the importance of prioritising immunisation as a safe and highly cost-effective intervention to prevent illness.^11^

The results of this study provide a starting point for discussions on research prioritisation among stakeholders working on Afghanistan’s communicable disease crisis, highlighting critical evidence gaps and some research that could be considered ‘low hanging fruit’. For example, simple and relatively inexpensive research could help to clarify the question of barriers to low immunisation coverage which could have implications for programme delivery or re-design. However, given the 14.5% response rate, 3-year lag between question generation and ranking, and exclusion of key stakeholder groups including the de facto authorities, these priorities should be validated through broader consultation before significant resource allocation decisions are made. Gavi, the Vaccine Alliance has historically supported Afghanistan in immunisation campaigns, but that funding has decreased significantly since 2021.^28^ With the 2025 shutdown of major US-based implementing partners and donors, such as USAID, the funding situation has deteriorated immensely. However, there is still support for communicable disease management in Afghanistan, such as a 2025 partnership with Japan to provide US$5 million to fight polio in Afghanistan.^29^ To effectively and powerfully impact the communicable disease crisis in Afghanistan with limited resources, researchers, the de facto authorities, donors, and programme implementers need to align on top priorities, as informed by this and other priority setting exercises. Scaling of effective priority interventions in this complex environment will then require government acceptance or NGO buy in and will, in all cases, depend on increased and sustained funding.

### Limitations

Many of the limitations relating to methods and processes summarised in the CHNRIs published within this Supplement also apply here.^11 12^ Of note, there were minimal inputs into study design by local researchers, with the exception of question generation. In terms of responses, selection bias is a major limitation of this exercise, as with other CHNRIs. Participants self-selected to generate communicable disease-related questions for the CHNRI and self-selected to respond to the CHNRI survey. There is some overlap in participants across CHNRI exercises that are published in this Supplement; this may have led to a somewhat artificial convergence of priorities, despite the different topic areas (eg, a focus on immunisation from the lens of the health system compared with communicable disease control). Due to logistical barriers and efforts to capture experienced researchers, there was no inclusion of the de facto authorities, frontline healthcare workers, or Afghan community members. These stakeholders may have scored questions differently, with exclusions potentially reinforcing externally driven agendas and risking a disconnect between CHNRI findings and the priorities of the Afghan community at large. We report a low survey response rate (14.5%) which can be partially explained by the lapse in time between initial contact with researchers and survey distribution, but may also reflect a reality of disengagement, burnout, or constrained participation for those working in complex and fragile settings such as Afghanistan. Additionally, there was over-representation by males (82%) and those working in academia/research (57%), underscoring a possible framing and ranking of research questions with unconscious bias and a lack of lived experience (for men) in a setting that is wholly unequal for women. Given differences in burden and access to care, it is plausible that Afghan women might have different priorities relating to communicable disease research. We note, also, that the diseases commonly prioritised within this exercise are the ones for which funding is mostly available and expertise exists; they do not necessarily align with the top causes of death in Afghanistan which include neonatal infections and lower respiratory infections. Critically, research questions were generated in 2022 but ranking occurred in 2025—a 3-year gap during which Afghanistan experienced fundamental political, economic, and health system transformations. The August 2021 regime change preceded question generation, but subsequent deterioration including: (1) the 2023–2024 massive reduction in international aid, (2) escalating measles outbreaks from 127 (2023) to 430 (2024), (3) the 2025 shutdown of USAID programming affecting hundreds of health facilities and (4) emergence of antimicrobial resistance as a leading mortality cause—all occurred after questions were formulated. This temporal disconnect raises concerns about whether the generated questions adequately capture current priorities. Respondents ranked questions that may have been outdated or incomplete, given evolving circumstances. Finally, Afghanistan faces worsening communicable disease challenges now, especially due to the 2025 change in the US presidential administration, which resulted in massive cuts in global aid to Afghanistan through programmes such as USAID. These shifts may have had significant implications for question generation and rankings.

## Conclusions

This application of the CHNRI methodology systematically and transparently identifies the top research priorities for communicable diseases in Afghanistan. The highest-ranked questions centred on identifying barriers to low vaccination coverage for measles and polio, assessing disease burden across regions, and reducing the incidence of TB. Top priority questions focused on immunisation, TB, and implementation challenges in a fragile health system, reflecting the need for operational and programmatic support through research. These findings present the beginning of an evidence-based roadmap for researchers, universities, governments and donors to align on approaches to control and reduce the spread of preventable and often fatal diseases in Afghanistan but require revalidation and scenario-based interpretation in the current Afghan context. This can help Afghanistan to undertake the required research to fill evidence gaps, design high impact evidence-based programmes to address priority health problems, and ultimately advance toward the United Nations SDGs.^11^

## Supplementary material

10.1136/bmjgh-2025-020891 online supplemental file 1

10.1136/bmjgh-2025-020891 online supplemental file 2

## Data Availability

All data relevant to the study are included in the article or uploaded as supplementary information.
