# Supplementary material for: Prioritising communicable disease research in Afghanistan: an application of the Child Health and Nutrition Research Initiative (CHNRI) methodology
Source: BMJ Glob Health. 2026 May 19;10(Suppl 3):e020891. doi: 10.1136/bmjgh-2025-020891 (PMC13202066; doi:10.1136/bmjgh-2025-020891)
Supplement: online supplemental file 1 [file bmjgh-10-Suppl_3-s001.docx]

**Supplemental Document**

Table of Contents

[Table 1. Afghanistan CHNRI Strategic Advisory Board Members. 1](#_Toc222746708)

[Table 2. CHNRI Criteria Options for Ranking by the Strategic Advisory Board. 2](#_Toc222746709)

[Table 3. Overall Ranking, Research Question, 4D’s Domain, Intermediate Research Priority Scores, Overall Research Priority Scores, and Average Expert Agreement for all scored research questions. 3](#_Toc222746710)

[Table 4. Overall Rank, Intermediate Research Priority Scores, and Overall Research Priority Scores for all research questions among Afghan respondents. 8](#_Toc222746711)

[Table 5. Overall Rank, Intermediate Research Priority Scores, and Overall Research Priority Scores for all research questions among non-Afghan respondents. 14](#_Toc222746712)

## **Table 1.** Afghanistan CHNRI Strategic Advisory Board Members.

| **SAB Member Name** | **Organization** | **Position Title** | **Health Topic Expertise** | **Number of Afghanistan Publications** |
| --- | --- | --- | --- | --- |
| Catherine Todd, MD, MPH | Pact | Senior Technical Advisor, Global Health | MNCH, HIV, SRH, mHealth | 45 |
| Hannah Tappis, MPH, DrPH | Jhpiego  Johns Hopkins University Bloomberg School of Public Health | Senior Measurement, Evaluation and Learning Advisor  Associate Faculty, Center for Humanitarian Health, Department of International Health | MNCH | 23 |
| David Peters, MD | York University, Faculty of Health | Dean | MNCH, Health Systems | 21 |
| Najibullah Safi, MD, MSc | World Health Organization | Program Manager, Health System Development | Health Systems, SRH, MNCH, Infectious Disease (COVID, Malaria) | 18 |
| Ahmad Shah Salehi, MD, MBA, MSc, PhD | Lapis Communications | Senior Public Health Advisor | MNCH, Health Systems | 12 |
| Kerri Wazny, PhD | Children’s Investment Fund Foundation | EME Manager - Nutrition | CHNRI Methodology |  |
| Mickey Chopra, MD, MPH, PhD | World Bank | Global Solutions Lead for Service Delivery | MNCH, Health Systems, Mental Health | 4 |
| Robert Black, MD, MPH | Johns Hopkins University Bloomberg School of Public Health | Director for the Institute of International Programs  Professor | Health Systems, MNCH, Nutrition  CHRNI Methodology |  |

## **Table 2.** CHNRI Criteria Options for Ranking by the Strategic Advisory Board.

| **Criterion** | **Question** |
| --- | --- |
| Feasibility | Is it feasible to conduct this research given security risks and other challenges in Afghanistan? |
| Respectfulness/Acceptability | Will the research and the results of the research be respectful and acceptable to local beliefs and cultural practices? |
| Affordability/Appropriateness of the Cost | Would the intervention evidenced by this research be affordable or cost effective?  Would this research be affordable/cost effective? |
| Answerability | How important is it for the researchers to be able to create a study to properly answer their research question? |
| Equity | Would the outcome of the research result in increased fairness in access to services in Afghanistan? |
| Disease Burden Reduction | Does this intervention/research address conditions which have a high burden in Afghanistan? |
| Effectiveness | Will the intervention/research results have the potential to impact people given the situational challenges experienced by Afghanistan? |
| Deliverability | Will the results of the research be able to be delivered and be affordable to the Afghan population or those who pay for the results (Afghan national/local governments)? |
| Likelihood to fill a knowledge gap | Will this research result in new information in Afghanistan? |
| Likelihood to attract donor attention | Will the result of this research eventually result in funding/attention from donors? |
| Implementation | Can the intervention or results of this research be changed to fit different groups of people (for example, different regions of the country, ethnic groups, urban vs. rural populations)? |
| Technical Possibility | If the research involves technology, will the technology be easy to use and not expensive to develop given the contextual limitations in Afghanistan? |
| Innovation | Will the results of the research make something better than what is currently being used in Afghanistan? |
| Sustainability | Will results of the research leave a long-lasting impact on the health of the Afghan population? |

## **Table 3.** Overall Ranking, Research Question, 4D’s Domain, Intermediate Research Priority Scores, Overall Research Priority Scores, and Average Expert Agreement for all scored research questions.

| **Ranking** | **Research Question** | **Domain** | **Feasibility** | **Effectiveness** | **Equity** | **Answerability** | **Disease Burden Reduction** | **Overall RPS** | **AEA** |
| --- | --- | --- | --- | --- | --- | --- | --- | --- | --- |
| 1 | *What are the barriers contributing to low polio and measles vaccination coverage among children in Afghanistan?* | Description | 0.90277779 | 0.91891891 | 0.93243241 | 0.95833331 | 0.93055558 | **92.86%** | 0.86 |
| 2 | *What are the current burdens of infectious diseases, including vaccine-preventable diseases (e.g., measles, polio, acute viral hepatitis, typhoid fever, pneumonia, meningitis), respiratory infections (e.g., tuberculosis, pneumonia,) and re-emerging diseases (e.g., acute watery diarrhea, measles, malaria), at the national and sub-national levels, and in rural areas of diarrhea, measles, malaria), at the national and sub-national levels, and in rural areas of Afghanistan?* | Description | 0.93902439 | 0.91463417 | 0.92857140 | 0.92857140 | 0.92307693 | **92.68%** | 0.89 |
| 3 | *What strategies should be considered to reduce the tuberculosis incidence in Afghanistan?* | Development | 0.953125 | 0.89393938 | 0.90625 | 0.92424244 | 0.875 | **91.05%** | 0.85 |
| 4 | *How does the misuse of antibiotics, including overprescribing and self-medicating, affect antibiotic resistance and the effectiveness of infectious disease control measures in Afghanistan?* | Description | 0.86363637 | 0.921875 | 0.859375 | 0.969697 | 0.90625 | **90.42%** | 0.87 |
| 5 | *What is the prevalence and associated morbidity and mortality of vaccine-preventable diseases among children in Afghanistan who are seeking health services from outpatient and inpatient departments at health facilities?* | Description | 0.93421054 | 0.89473683 | 0.87179488 | 0.89473683 | 0.91891891 | **90.29%** | 0.84 |
| 6 | *What is the status of the immunization program and how can program performance be improved to increase coverage under the new regime?* |  | 0.890625 | 0.90322578 | 0.90322578 | 0.88709676 | 0.91935486 | **90.07%** | 0.81 |
| 7 | *What are the determinants of the higher prevalence of tuberculosis among women in Afghanistan compared to men?* | Description | 0.84375 | 0.90625 | 0.9375 | 0.89393938 | 0.921875 | **90.07%** | 0.81 |
| 8 | *How can immunization programs and other strategies for reducing the prevalence and mortality of communicable and infectious diseases be effectively delivered across Afghanistan?* | Delivery | 0.91666669 | 0.89999998 | 0.90277779 | 0.875 | 0.87142855 | **89.32%** | 0.83 |
| 9 | *What are the prevalence and risk factors of blood-borne diseases (e.g., hepatitis B, hepatitis C, HIV/AIDS) at the national level in Afghanistan and among high-risk populations such as people who inject drugs, people living under the poverty line, the unemployed, and internally displaced people?* | Description | 0.80000001 | 0.92500001 | 0.93902439 | 0.89999998 | 0.88157892 | **88.91%** | 0.82 |
| 10 | *What are the most effective methods for identifying areas in Afghanistan with low polio and measles vaccination coverage?* | Development | 0.875 | 0.92105263 | 0.84210527 | 0.9054054 | 0.89999998 | **88.87%** | 0.83 |
| 11 | *How can leishmaniasis cases best be identified and followed-up at the community level in Afghanistan?* | Discovery | 0.890625 | 0.875 | 0.88333333 | 0.95161289 | 0.83870965 | **88.79%** | 0.81 |
| 12 | *What are the reasons for the underutilization of long-lasting insecticide-treated bednets (LLINs) among people at risk of malaria in Afghanistan?* | Description | 0.90322578 | 0.90322578 | 0.90322578 | 0.921875 | 0.75 | **87.63%** | 0.79 |
| 13 | *What factors have contributed to the plateauing of tuberculosis incidence in Afghanistan?* | Delivery | 0.90625 | 0.859375 | 0.84375 | 0.875 | 0.82258064 | **86.14%** | 0.77 |
| 14 | *What are the prevalence and risk factors of malaria and neglected tropical diseases (e.g., helminths, toxoplasmosis, kala-azar, ascariasis, and other water-, soil- and food-borne infections) in Afghanistan and among vulnerable populations including mothers and children?* | Description | 0.87837839 | 0.86486489 | 0.85526317 | 0.8888889 | 0.81944442 | **86.14%** | 0.79 |
| 15 | *What is the burden of tuberculosis and the level of access to control measures among high-risk populations in Afghanistan (e.g., people living with mental illness and drug users), and what approaches can be considered to improve outcomes for these groups?* | Description, Development | 0.796875 | 0.88709676 | 0.88333333 | 0.796875 | 0.88709676 | **85.03%** | 0.74 |
| 16 | *How can local education campaigns be designed and implemented to address vaccine hesitancy in Afghanistan?* | Development | 0.87878788 | 0.82352942 | 0.82352942 | 0.88235295 | 0.81818181 | **84.53%** | 0.76 |
| 17 | *What are the immediate and sustainable options for improving hygiene practices and preventing diarrhea illnesses, including cholera, at the national level in Afghanistan and among vulnerable populations, such as internally displaced persons?* | Delivery | 0.87142855 | 0.82857144 | 0.84285712 | 0.85714287 | 0.82352942 | **84.47%** | 0.78 |
| 18 | *What risk assessment criteria could be used to predict different outbreaks in Afghanistan considering population immunities, geographical locations, residential characteristics, and socioeconomic status?* | Development | 0.82352942 | 0.86363637 | 0.82352942 | 0.86764705 | 0.84375 | **84.44%** | 0.74 |
| 19 | *What is the current state of vaccine hesitancy in Afghanistan, and what are the key barriers and facilitators to vaccination?* | Description | 0.90909094 | 0.81818181 | 0.83333331 | 0.88235295 | 0.765625 | **84.17%** | 0.76 |
| 20 | *What are the effective communication strategies to increase the utilization of long-lasting insecticide-treated bednets (LLLINs) among people at risk of malaria in Afghanistan?* | Development | 0.83870965 | 0.80645162 | 0.84482759 | 0.87096775 | 0.83333331 | **83.89%** | 0.73 |
| 21 | *How aware are school-aged children, high school students, and university students in Afghanistan of the risks and protective measures related to highly prevalent communicable diseases, such as HIV/AIDS and hepatitis?* | Description | 0.84482759 | 0.85000002 | 0.90625 | 0.88709676 | 0.66216218 | **83.01%** | 0.79 |
| 22 | *What are the effects of population displacement in Afghanistan on the global spread of infections?* | Description | 0.828125 | 0.81666666 | 0.75806451 | 0.91935486 | 0.78333336 | **82.11%** | 0.79 |
| 23 | *What actions can be taken at the community level to reduce the stigma associated with leishmaniasis in Afghanistan?* | Delivery | 0.89393938 | 0.77272725 | 0.828125 | 0.90909094 | 0.67741936 | **81.63%** | 0.73 |
| 24 | *What are effective media campaigns that can be implemented at the community level to reduce the stigma of leishmaniasis in Afghanistan?* | Delivery, Development | 0.77419353 | 0.84375 | 0.75806451 | 0.875 | 0.75 | **80.02%** | 0.71 |
| 25 | *How can vaccine-preventable diseases, including wild polio, be effectively surveilled and controlled in the border areas between Afghanistan and Pakistan?* | Development | 0.75757575 | 0.8142857 | 0.75714284 | 0.75714284 | 0.75757575 | **76.87%** | 0.64 |
| 26 | *What are the bionomics of malaria vectors in different areas of Afghanistan with distinct malaria risk levels?* | Description | 0.76666665 | 0.72580647 | 0.765625 | 0.8125 | 0.76666665 | **76.75%** | 0.63 |
| 27 | *What is the current COVID-19 seroprevalence, vaccine coverage, and related mortality in Afghanistan?* | Description | 0.78787881 | 0.72058821 | 0.77941179 | 0.81944442 | 0.703125 | **76.21%** | 0.70 |
| 28 | *What is the prevalence and what are the most common clinical symptoms of congenital toxoplasmosis among infants in Laghman and Nangarhar provinces of Afghanistan?* | Description | 0.82692307 | 0.7758621 | 0.76785713 | 0.75862068 | 0.64285713 | **75.44%** | 0.64 |
| 29 | *What is the prevalence of acute Toxoplasma gondii infection among pregnant women in Laghman and Nangarhar provinces of Afghanistan, and what are the most common strains and clinical symptoms?* | Description | 0.76785713 | 0.76785713 | 0.74137932 | 0.7758621 | 0.63793105 | **73.82%** | 0.63 |
| 30 | *How can the existing COVID-19 molecular reference laboratories in Afghanistan be utilized for additional purposes, such as genome surveillance, local production of molecular diagnostic kits, and detection of other diseases, including measles, leishmaniasis, malaria, poliovirus infection, and genetic disorders?* | Development | 0.7241379 | 0.75 | 0.7096774 | 0.75 | 0.65625 | **71.80%** | 0.61 |
| 31 | *What is the impact of the political transition in Afghanistan on delivering health services for infectious diseases, including infection prevention and control programs?* | Description | 0.74242425 | 0.71428573 | 0.69999999 | 0.72058821 | 0.66176468 | **70.78%** | 0.56 |
| 32 | *How does acute Toxoplasma gondii infection impact the incidence of abortion in Langhman and Nangarhar provinces in Afghanistan?* | Description | 0.68000001 | 0.7037037 | 0.62962961 | 0.64285713 | 0.53448278 | **63.81%** | 0.53 |
| 33 | *What is the current state of research on arboviruses and their vectors with a high risk of causing epidemics in Afghanistan, particularly studies using DNA-based methods?* | Description | 0.60714287 | 0.5535714 | 0.5 | 0.60714287 | 0.51724136 | **55.70%** | 0.43 |

## **Table 4.** Overall Rank, Intermediate Research Priority Scores, and Overall Research Priority Scores for all research questions among Afghan respondents.

| **Ranking** | **Research Question** | **Feasibility** | **Effectiveness** | **Equity** | **Answerability** | **Disease Burden Reduction** | **Overall RPS** |
| --- | --- | --- | --- | --- | --- | --- | --- |
| 1 | *How does the misuse of antibiotics, including overprescribing and self-medicating, affect antibiotic resistance and the effectiveness of infectious disease control measures in Afghanistan?* | 0.9285714 | 1 | 0.925 | 1 | 0.952381 | **96.12%** |
| 2 | *What are the barriers contributing to low polio and measles vaccination coverage among children in Afghanistan?* | 0.95454544 | 0.95652175 | 0.95652175 | 0.97826087 | 0.95454544 | **96.01%** |
| 3 | *What are the determinants of the higher prevalence of tuberculosis among women in Afghanistan compared to men?* | 0.90476191 | 0.9285714 | 0.92500001 | 0.9285714 | 0.97500002 | **93.24%** |
| 4 | *How can immunization programs and other strategies for reducing the prevalence and mortality of communicable and infectious diseases be effectively delivered across Afghanistan?* | 0.95454544 | 0.9285714 | 0.93181819 | 0.90909094 | 0.9285714 | **93.05%** |
| 5 | *What is the status of the immunization program and how can program performance be improved to increase coverage under the new regime?* | 0.88095236 | 0.97500002 | 0.92500001 | 0.92500001 | 0.92500001 | **92.62%** |
| 6 | *What is the prevalence and associated morbidity and mortality of vaccine-preventable diseases among children in Afghanistan who are seeking health services from outpatient and inpatient departments at health facilities?* | 0.97916669 | 0.89999998 | 0.88 | 0.92000002 | 0.9375 | **92.33%** |
| 7 | *What are the prevalence and risk factors of blood-borne diseases (e.g., hepatitis B, hepatitis C, HIV/AIDS) at the national level in Afghanistan and among high-risk populations such as people who inject drugs, people living under the poverty line, the unemployed, and internally displaced people?* | 0.84615386 | 0.96153843 | 0.94230771 | 0.94230771 | 0.89999998 | **91.85%** |
| 8 | *What are the current burdens of infectious diseases, including vaccine-preventable diseases (e.g., measles, polio, acute viral hepatitis, typhoid fever, pneumonia, meningitis), respiratory infections (e.g., tuberculosis, pneumonia,) and re-emerging diseases (e.g., acute watery diarrhea, measles, malaria), at the national and sub-national levels, and in rural areas of diarrhea, measles, malaria), at the national and sub-national levels, and in rural areas of Afghanistan?* | 0.8888889 | 0.92592591 | 0.9074074 | 0.94444442 | 0.92000002 | **91.73%** |
| 9 | *How can leishmaniasis cases best be identified and followed-up at the community level in Afghanistan?* | 0.92500001 | 0.89999998 | 0.91666669 | 0.94736844 | 0.89473683 | **91.68%** |
| 10 | *What strategies should be considered to reduce the tuberculosis incidence in Afghanistan?* | 0.97500002 | 0.88095236 | 0.85714287 | 0.90476191 | 0.875 | **89.86%** |
| 11 | *What are the reasons for the underutilization of long-lasting insecticide-treated bednets (LLINs) among people at risk of malaria in Afghanistan?* | 0.92105263 | 0.94736844 | 0.92105263 | 0.89999998 | 0.77777779 | **89.35%** |
| 12 | *What are the most effective methods for identifying areas in Afghanistan with low polio and measles vaccination coverage?* | 0.86956519 | 0.9375 | 0.79166669 | 0.91666669 | 0.90909094 | **88.49%** |
| 13 | *How aware are school-aged children, high school students, and university students in Afghanistan of the risks and protective measures related to highly prevalent communicable diseases, such as HIV/AIDS and hepatitis?* | 0.86842108 | 0.88095236 | 0.97619045 | 0.94999999 | 0.70833331 | **87.68%** |
| 14 | *What factors have contributed to the plateauing of tuberculosis incidence in Afghanistan?* | 0.90476191 | 0.90476191 | 0.85000002 | 0.85000002 | 0.86842108 | **87.56%** |
| 15 | *What risk assessment criteria could be used to predict different outbreaks in Afghanistan considering population immunities, geographical locations, residential characteristics, and socioeconomic status?* | 0.88095236 | 0.90476191 | 0.83333331 | 0.88095236 | 0.875 | **87.50%** |
| 16 | *How can local education campaigns be designed and implemented to address vaccine hesitancy in Afghanistan?* | 0.85000002 | 0.83333331 | 0.85714287 | 0.90476191 | 0.89999998 | **86.90%** |
| 17 | *What is the current state of vaccine hesitancy in Afghanistan, and what are the key barriers and facilitators to vaccination?* | 0.875 | 0.875 | 0.875 | 0.85714287 | 0.84210527 | **86.48%** |
| 18 | *What are the immediate and sustainable options for improving hygiene practices and preventing diarrhea illnesses, including cholera, at the national level in Afghanistan and among vulnerable populations, such as internally displaced persons?* | 0.85714287 | 0.83333331 | 0.85714287 | 0.88095236 | 0.875 | **86.07%** |
| 19 | *What are the prevalence and risk factors of malaria and neglected tropical diseases (e.g., helminths, toxoplasmosis, kala-azar, ascariasis, and other water-, soil- and food-borne infections) in Afghanistan and among vulnerable populations including mothers and children?* | 0.85416669 | 0.89583331 | 0.83333331 | 0.85416669 | 0.84090906 | **85.57%** |
| 20 | *What is the burden of tuberculosis and the level of access to control measures among high-risk populations in Afghanistan (e.g., people living with mental illness and drug users), and what approaches can be considered to improve outcomes for these groups?* | 0.83333331 | 0.85000002 | 0.85000002 | 0.80952382 | 0.89999998 | **84.86%** |
| 21 | *What are the effective communication strategies to increase the utilization of long-lasting insecticide-treated bednets (LLLINs) among people at risk of malaria in Afghanistan?* | 0.84210527 | 0.81578946 | 0.85294116 | 0.84210527 | 0.83333331 | **83.73%** |
| 22 | *What actions can be taken at the community level to reduce the stigma associated with leishmaniasis in Afghanistan?* | 0.9285714 | 0.76190478 | 0.80952382 | 0.90476191 | 0.65789473 | **81.25%** |
| 23 | *What are effective media campaigns that can be implemented at the community level to reduce the stigma of leishmaniasis in Afghanistan?* | 0.82499999 | 0.85000002 | 0.71052629 | 0.85000002 | 0.75 | **79.71%** |
| 24 | *What are the effects of population displacement in Afghanistan on the global spread of infections?* | 0.78571427 | 0.80000001 | 0.71428573 | 0.875 | 0.7631579 | **78.76%** |
| 25 | *How can vaccine-preventable diseases, including wild polio, be effectively surveilled and controlled in the border areas between Afghanistan and Pakistan?* | 0.80000001 | 0.78571427 | 0.76190478 | 0.71428573 | 0.78947371 | **77.03%** |
| 26 | *What is the prevalence of acute Toxoplasma gondii infection among pregnant women in Laghman and Nangarhar provinces of Afghanistan, and what are the most common strains and clinical symptoms?* | 0.8888889 | 0.7368421 | 0.68421054 | 0.7631579 | 0.68421054 | **75.15%** |
| 27 | *What are the bionomics of malaria vectors in different areas of Afghanistan with distinct malaria risk levels?* | 0.72500002 | 0.71428573 | 0.69047618 | 0.76190478 | 0.75 | **72.83%** |
| 28 | *What is the current COVID-19 seroprevalence, vaccine coverage, and related mortality in Afghanistan?* | 0.73809522 | 0.69047618 | 0.76190478 | 0.75 | 0.68421054 | **72.49%** |
| 29 | *What is the prevalence and what are the most common clinical symptoms of congenital toxoplasmosis among infants in Laghman and Nangarhar provinces of Afghanistan?* | 0.8125 | 0.7368421 | 0.68421054 | 0.71052629 | 0.66666669 | **72.21%** |
| 30 | *What is the impact of the political transition in Afghanistan on delivering health services for infectious diseases, including infection prevention and control programs?* | 0.67500001 | 0.75 | 0.72727275 | 0.64285713 | 0.73809522 | **70.66%** |
| 31 | *How can the existing COVID-19 molecular reference laboratories in Afghanistan be utilized for additional purposes, such as genome surveillance, local production of molecular diagnostic kits, and detection of other diseases, including measles, leishmaniasis, malaria, poliovirus infection, and genetic disorders?* | 0.68421054 | 0.75 | 0.68421054 | 0.72500002 | 0.68421054 | **70.55%** |
| 32 | *How does acute Toxoplasma gondii infection impact the incidence of abortion in Langhman and Nangarhar provinces in Afghanistan?* | 0.75 | 0.71052629 | 0.60526317 | 0.63157892 | 0.52499998 | **64.45%** |
| 33 | *What is the current state of research on arboviruses and their vectors with a high risk of causing epidemics in Afghanistan, particularly studies using DNA-based methods?* | 0.44117647 | 0.5 | 0.41666666 | 0.5 | 0.44444445 | **46.05%** |

## **Table 5.** Overall Rank, Intermediate Research Priority Scores, and Overall Research Priority Scores for all research questions among non-Afghan respondents.

| **Ranking** | **Research Question** | **Feasibility** | **Effectiveness** | **Equity** | **Answerability** | **Disease Burden Reduction** | **Overall RPS** |
| --- | --- | --- | --- | --- | --- | --- | --- |
| 1 | *What strategies should be considered to reduce the tuberculosis incidence in Afghanistan?* | 0.91666669 | 0.91666669 | 1 | 0.95833331 | 0.875 | **93.33%** |
| 2 | *What are the current burdens of infectious diseases, including vaccine-preventable diseases (e.g., measles, polio, acute viral hepatitis, typhoid fever, pneumonia, meningitis), respiratory infections (e.g., tuberculosis, pneumonia,) and re-emerging diseases (e.g., acute watery diarrhea, measles, malaria), at the national and sub-national levels, and in rural areas of diarrhea, measles, malaria), at the national and sub-national levels, and in rural areas of Afghanistan?* | 0.96428573 | 0.8928571 | 0.96666664 | 0.89999998 | 0.9285714 | **93.05%** |
| 3 | *What are the most effective methods for identifying areas in Afghanistan with low polio and measles vaccination coverage?* | 0.88461536 | 0.89285713 | 0.9285714 | 0.88461536 | 0.88461536 | **89.51%** |
| 4 | *What are the effects of population displacement in Afghanistan on the global spread of infections?* | 0.90909094 | 0.85000002 | 0.85000002 | 1 | 0.81818181 | **88.55%** |
| 5 | *What are the barriers contributing to low polio and measles vaccination coverage among children in Afghanistan?* | 0.8214286 | 0.85714287 | 0.89285713 | 0.92307693 | 0.89285713 | **87.75%** |
| 6 | *What are the prevalence and risk factors of malaria and neglected tropical diseases (e.g., helminths, toxoplasmosis, kala-azar, ascariasis, and other water-, soil- and food-borne infections) in Afghanistan and among vulnerable populations including mothers and children?* | 0.92307693 | 0.80769229 | 0.89285713 | 0.95833331 | 0.78571427 | **87.35%** |
| 7 | *What is the prevalence and associated morbidity and mortality of vaccine-preventable diseases among children in Afghanistan who are seeking health services from outpatient and inpatient departments at health facilities?* | 0.85714287 | 0.88461536 | 0.85714287 | 0.84615386 | 0.88461536 | **86.59%** |
| 8 | *What is the status of the immunization program and how can program performance be improved to increase coverage under the new regime?* | 0.90909094 | 0.77272725 | 0.86363637 | 0.81818181 | 0.90909094 | **85.45%** |
| 9 | *What is the burden of tuberculosis and the level of access to control measures among high-risk populations in Afghanistan (e.g., people living with mental illness and drug users), and what approaches can be considered to improve outcomes for these groups?* | 0.72727275 | 0.95454544 | 0.94999999 | 0.77272725 | 0.86363637 | **85.36%** |
| 10 | *What are the reasons for the underutilization of long-lasting insecticide-treated bednets (LLINs) among people at risk of malaria in Afghanistan?* | 0.875 | 0.83333331 | 0.875 | 0.95833331 | 0.70833331 | **85.00%** |
| 11 | *What are the bionomics of malaria vectors in different areas of Afghanistan with distinct malaria risk levels?* | 0.85000002 | 0.75 | 0.90909094 | 0.90909094 | 0.80000001 | **84.36%** |
| 12 | *What are the determinants of the higher prevalence of tuberculosis among women in Afghanistan compared to men?* | 0.72727275 | 0.86363637 | 0.95833331 | 0.83333331 | 0.83333331 | **84.32%** |
| 13 | *What are the effective communication strategies to increase the utilization of long-lasting insecticide-treated bednets (LLLINs) among people at risk of malaria in Afghanistan?* | 0.83333331 | 0.79166669 | 0.83333331 | 0.91666669 | 0.83333331 | **84.17%** |
| 14 | *How can leishmaniasis cases best be identified and followed-up at the community level in Afghanistan?* | 0.83333331 | 0.83333331 | 0.83333331 | 0.95833331 | 0.75 | **84.17%** |
| 15 | *What factors have contributed to the plateauing of tuberculosis incidence in Afghanistan?* | 0.90909094 | 0.77272725 | 0.83333331 | 0.91666669 | 0.75 | **83.64%** |
| 16 | *How can immunization programs and other strategies for reducing the prevalence and mortality of communicable and infectious diseases be effectively delivered across Afghanistan?* | 0.85714287 | 0.85714287 | 0.85714287 | 0.8214286 | 0.78571427 | **83.57%** |
| 17 | *What are the prevalence and risk factors of blood-borne diseases (e.g., hepatitis B, hepatitis C, HIV/AIDS) at the national level in Afghanistan and among high-risk populations such as people who inject drugs, people living under the poverty line, the unemployed, and internally displaced people?* | 0.71428573 | 0.85714287 | 0.93333334 | 0.8214286 | 0.84615386 | **83.45%** |
| 18 | *What is the current COVID-19 seroprevalence, vaccine coverage, and related mortality in Afghanistan?* | 0.875 | 0.76923078 | 0.80769229 | 0.95833331 | 0.73076922 | **82.82%** |
| 19 | *What actions can be taken at the community level to reduce the stigma associated with leishmaniasis in Afghanistan?* | 0.83333331 | 0.79166669 | 0.86363637 | 0.91666669 | 0.70833331 | **82.27%** |
| 20 | *What are the immediate and sustainable options for improving hygiene practices and preventing diarrhea illnesses, including cholera, at the national level in Afghanistan and among vulnerable populations, such as internally displaced persons?* | 0.89285713 | 0.8214286 | 0.8214286 | 0.8214286 | 0.75 | **82.14%** |
| 21 | *What is the prevalence and what are the most common clinical symptoms of congenital toxoplasmosis among infants in Laghman and Nangarhar provinces of Afghanistan?* | 0.85000002 | 0.85000002 | 0.94444442 | 0.85000002 | 0.60000002 | **81.89%** |
| 22 | *How can local education campaigns be designed and implemented to address vaccine hesitancy in Afghanistan?* | 0.92307693 | 0.80769229 | 0.76923078 | 0.84615386 | 0.69230771 | **80.77%** |
| 23 | *What is the current state of vaccine hesitancy in Afghanistan, and what are the key barriers and facilitators to vaccination?* | 0.96153843 | 0.73076922 | 0.76923078 | 0.92307693 | 0.65384614 | **80.77%** |
| 24 | *What are effective media campaigns that can be implemented at the community level to reduce the stigma of leishmaniasis in Afghanistan?* | 0.68181819 | 0.83333331 | 0.83333331 | 0.91666669 | 0.75 | **80.30%** |
| 25 | *How does the misuse of antibiotics, including overprescribing and self-medicating, affect antibiotic resistance and the effectiveness of infectious disease control measures in Afghanistan?* | 0.75 | 0.77272725 | 0.75 | 0.91666669 | 0.81818181 | **80.15%** |
| 26 | *What risk assessment criteria could be used to predict different outbreaks in Afghanistan considering population immunities, geographical locations, residential characteristics, and socioeconomic status?* | 0.73076922 | 0.79166669 | 0.80769229 | 0.84615386 | 0.79166669 | **79.36%** |
| 27 | *How can vaccine-preventable diseases, including wild polio, be effectively surveilled and controlled in the border areas between Afghanistan and Pakistan?* | 0.69230771 | 0.85714287 | 0.75 | 0.8214286 | 0.71428573 | **76.70%** |
| 28 | *How can the existing COVID-19 molecular reference laboratories in Afghanistan be utilized for additional purposes, such as genome surveillance, local production of molecular diagnostic kits, and detection of other diseases, including measles, leishmaniasis, malaria, poliovirus infection, and genetic disorders?* | 0.80000001 | 0.75 | 0.75 | 0.79166669 | 0.61538464 | **74.14%** |
| 29 | *How aware are school-aged children, high school students, and university students in Afghanistan of the risks and protective measures related to highly prevalent communicable diseases, such as HIV/AIDS and hepatitis?* | 0.80000001 | 0.77777779 | 0.77272725 | 0.77272725 | 0.57692307 | **74.00%** |
| 30 | *What is the current state of research on arboviruses and their vectors with a high risk of causing epidemics in Afghanistan, particularly studies using DNA-based methods?* | 0.86363637 | 0.64999998 | 0.64999998 | 0.80000001 | 0.63636363 | **72.00%** |
| 31 | *What is the prevalence of acute Toxoplasma gondii infection among pregnant women in Laghman and Nangarhar provinces of Afghanistan, and what are the most common strains and clinical symptoms?* | 0.55000001 | 0.83333331 | 0.85000002 | 0.80000001 | 0.55000001 | **71.67%** |
| 32 | *What is the impact of the political transition in Afghanistan on delivering health services for infectious diseases, including infection prevention and control programs?* | 0.84615386 | 0.65384614 | 0.65384614 | 0.84615386 | 0.53846157 | **70.77%** |
| 33 | *How does acute Toxoplasma gondii infection impact the incidence of abortion in Langhman and Nangarhar provinces in Afghanistan?* | 0.55555558 | 0.6875 | 0.6875 | 0.66666669 | 0.55555558 | **63.06%** |
