# Supplementary material for: Prioritising communicable disease research in Afghanistan: an application of the Child Health and Nutrition Research Initiative (CHNRI) methodology
Source: BMJ Glob Health. 2026 May 19;10(Suppl 3):e020891. doi: 10.1136/bmjgh-2025-020891 (PMC13202066; doi:10.1136/bmjgh-2025-020891)
Supplement: online supplemental file 2 [file bmjgh-10-Suppl_3-s002.docx]

### BMJ Global Health Author Reflexivity Statement

Adapted from Morton, B., Vercueil, A., Masekela, R., Heinz, E., Reimer, L., Saleh, S., Kalinga, C., Seekles, M., Biccard, B., Chakaya, J., Abimbola, S., Obasi, A. and Oriyo, N. (2022), Consensus statement on measures to promote equitable authorship in the publication of research from international partnerships. Anaesthesia, 77: 264-276. <https://doi.org/10.1111/anae.15597>

| **Study conceptualisation** | |
| --- | --- |
| 1. How does this study address local research and policy priorities? | The goal of this study was to use a crowdsourcing approach to prioritize research questions related to communicable disease management in Afghanistan, in order to ultimately improve the health and wellbeing of Afghans. To do so, Afghanistan health researchers were identified and asked to submit relevant research questions that were then scored and ranked. As researchers had to meet certain criteria (relating to having expertise in health research in Afghanistan), questions submitted for ranking were contextually/locally relevant. |
| 1. How were local researchers involved in study design? | Researchers – some of whom were local to Afghanistan – were involved in the submission of research questions. These questions then formed the basis of the CHNRI survey. One could argue that submitted research questions, which are then sent out through the survey for ranking, are the most critical design element of a CHNRI exercise. Without appropriate local and expert input into the development of questions that are specific to the Afghan context, then results will be somewhat arbitrary and meaningless. Other design elements (e.g., selection of CHNRI criteria and weighting) were decided in collaboration with the study Strategic Advisory Board, which consisted of Afghan health research experts including two Afghan-origin members. In terms of the other components of study design – data collection and data analysis – this was conducted solely by the core team at Johns Hopkins University (JHU), with required inputs by all co-authors related to aggregate data and findings at the review stage.  It should also be noted that the corresponding author is herself of Afghan origin; she visits the country often and has family there, in addition to conducting research in this setting. |
| **Research management** | |
| 1. How has funding been used to support the local research team(s)? | Funding for this study was mostly used to support publication and research management by the core JHU study team that did not include any local Afghan collaborators. Some Afghan researchers were part of the Strategic Advisory Board (though this was an unpaid, technical advisory role). CHNRI respondents were not paid for their time. |
| **Data acquisition and analysis** | |
| 1. How are research staff who conducted data collection acknowledged? | Data was collected through REDCap and was managed at JHU. |
| 1. How have members of the research partnership been provided with access to study data? | Only the core research team at JHU had access to the raw data, however, the data could be de-identified and shared upon request. The Strategic Advisory Board and all collaborators reviewed the aggregated study results. |
| 1. How were data used to develop analytical skills within the partnership? | Data analysis was conducted by the core team at JHU. |
| **Data interpretation** | |
| 1. How have research partners collaborated in interpreting study data? | All co-authors reviewed the aggregate study results and provided input into results interpretation. The manuscript was revised several times based on these inputs. |
| **Drafting and revising for intellectual content** | |
| 1. How were research partners supported to develop writing skills? | The core team at JHU drafted the initial manuscript, but all co-authors reviewed all drafts and provided feedback and/or directly edited the paper. Being a part of this process may have indirectly supported the development of writing skills. |
| 1. How will research products be shared to address local needs? | The research findings will be published as open-access and are aimed at instigating additional funding for priority communicable disease research and programming in Afghanistan. |
| **Authorship** | |
| 1. How is the leadership, contribution and ownership of this work by LMIC researchers recognised within the authorship? | All CHRNI respondents and the Strategic Advisory Board members were provided the opportunity for co-authorship. |
| 1. How have early career researchers across the partnership been included within the authorship team? | Most of the core team at JHU is comprised of early to mid-career researchers (ECK, TM, CK, SEB, HT, NA). |
| 1. How has gender balance been addressed within the authorship? | Within the main author by-line there are 8 females and 3 males. |
| **Training** | |
| 1. How has the project contributed to training of LMIC researchers? | N/A |
| **Infrastructure** | |
| 1. How has the project contributed to improvements in local infrastructure? | N/A |
| **Governance** | |
| 1. What safeguarding procedures were used to protect local study participants and researchers? | Respondents self-selected to participate in the survey, and all reported results have been anonymized and aggregated. Respondents were also invited to be named or un-named co-authors on the manuscript. |
